# A Nanobody‐LNP Platform for Targeting and Relicensing Dendritic Cells for Potent Cancer Immunotherapy

**DOI:** 10.1002/advs.202523024

**Published:** 2026-05-04

**Authors:** Shugang Qin, Zhiying Huang, Hai Huang, Yupei Zhang, Yuting Chen, Xi He, Zhenyi Niu, Zhongzheng Xiang, Chaoyu Zou, Xiangrong Song

**Affiliations:** ^1^ Department of Critical Care Medicine Frontiers Science Center for Disease‐related Molecular Network State Key Laboratory of Biotherapy and Cancer Center West China Hospital Sichuan University Chengdu China; ^2^ Department of Experimental Research Sichuan Clinical Research Center for Cancer Sichuan Cancer Center Sichuan Cancer Hospital & Institute University of Electronic Science and Technology of China Chengdu China; ^3^ Department of Head and Neck Surgery, Sichuan Clinical Research Center for Cancer, Sichuan Cancer Hospital & Institute, Sichuan Cancer Center University of Electronic Science and Technology of China Chengdu China

**Keywords:** anti‐tumor immune response, mRNA delivery system, mRNA vaccines, nanobody, targeting dendritic cells

## Abstract

Effective cancer immunotherapy requires not only efficient antigen delivery to dendritic cells (DCs) but also overcoming local immunosuppression. Here, we introduce a nanobody‐LNP platform that achieves both targeting and active relicensing of DCs. By decorating lipid nanoparticles with nanobodies against the DC surface protein Plastin‐2 (PLS2), our platform achieves a remarkable 93% internalization efficiency. This preferential targeting dramatically enhances antigen expression while simultaneously relicensing DCs toward a more potent, mature phenotype by inhibiting the immunosuppressive Leptin‐JAK2‐STAT3 signaling pathway. This integrated strategy unleashed potent cytotoxic T lymphocyte responses and led to marked inhibition of established tumors. Our work establishes PLS2 as a novel immunomodulatory receptor and presents a dual‐action delivery platform that significantly boosts cancer vaccine potency.

## Graphical abstract

Plastin‐2 (PLS2) is identified as a dual‐function receptor on DCs that mediates both nanoparticle uptake and immunomodulation. A nanobody‐LNP platform is engineered to integrate antigen delivery with relicensing DCs. The therapeutic strategy elicits potent anti‐tumor T cell responses and leads to significant inhibition of established tumors in vivo.

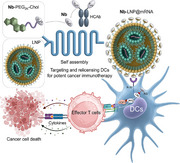

## 1 Introduction

With advantages such as rapid development and robust immune induction, mRNA vaccines are promising tools for cancer immunotherapy [1, 2]. Despite ongoing clinical trials, current mRNA cancer vaccines, often co‐administered with checkpoint inhibitors, show limited objective response rates (ORR), partly due to suboptimal biodistribution [3, 4, 5]. Vaccine efficacy hinges on efficient uptake and antigen presentation by professional antigen‐presenting cells (APCs), particularly dendritic cells (DCs) [6, 7]. Therefore, developing mRNA delivery platforms that effectively target DCs in vivo is critical for enhancing vaccine immunogenicity and therapeutic outcomes [8, 9].

Active targeting strategies modify nanoparticles with ligands such as carbohydrates [10, 11], peptides [12, 13, 14], antibodies [15, 16], and nanobodies [17]. Nanobodies (Nbs), small (12–15 kDa) and stable antigen‐binding fragments, offer distinct advantages for nanoparticle modification due to their high affinity and ease of conjugation [18]. Given the proven clinical utility of lipid nanoparticles (LNPs) for mRNA delivery [19, 20, 21, 22, 23], modifying LNPs with DC‐specific Nbs represents an attractive and largely underexplored strategy.

**FIGURE 1:**
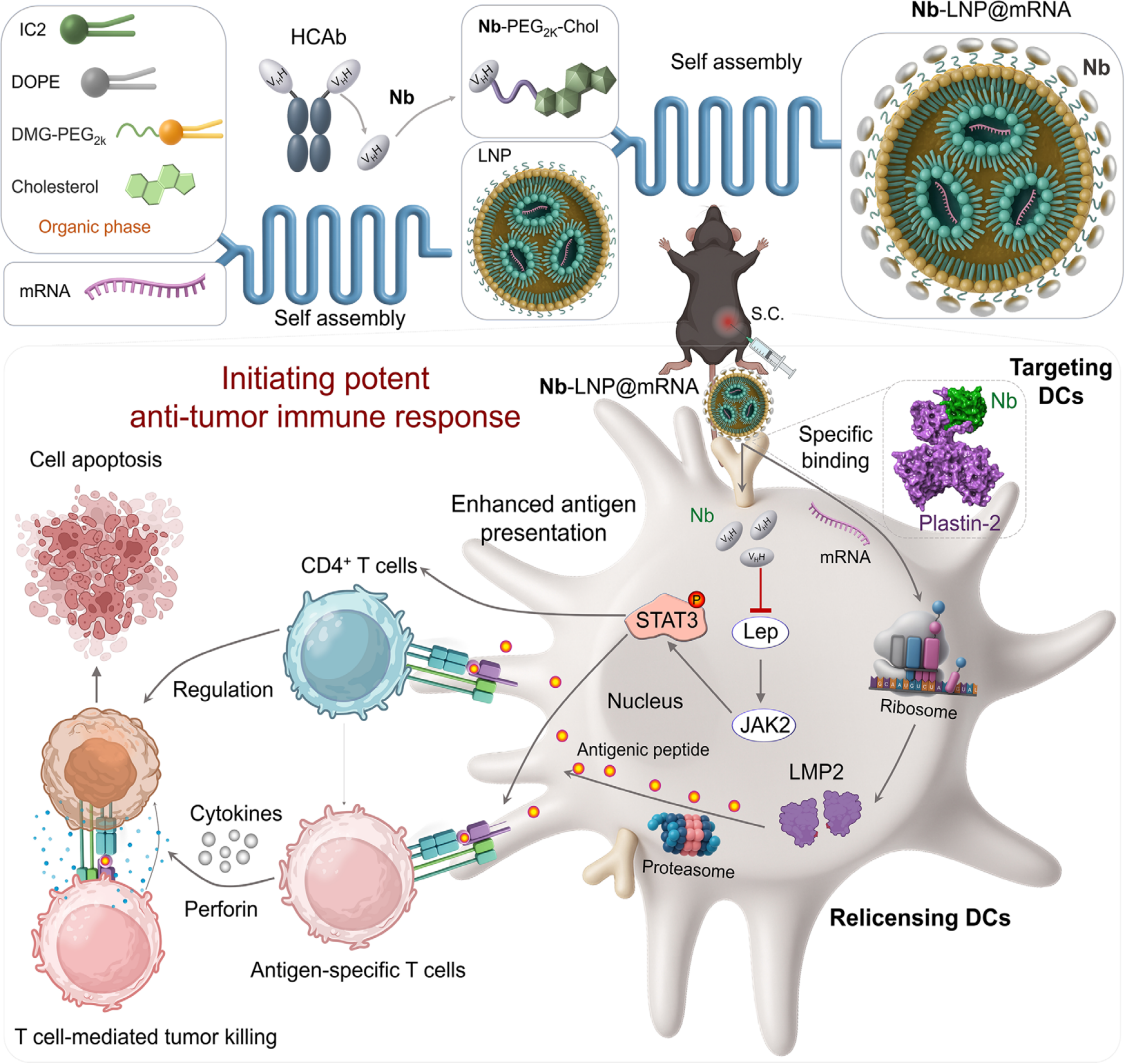
Nanobody‐modified mRNA nanovaccine targeting dendritic cells for anti‐tumor immunity. Our study developed an mRNA nanovaccine (Nb‐LNP@mRNA) modified with the nanobody (Nb, DC2.1), the antigen‐binding domain of a heavy‐chain‐only antibody (HCAb), designed to specifically target the transmembrane protein PLS2 on DCs. We prepared the LNP@mRNA by combining an organic phase (IC2, cholesterol, DOPE, and DMG‐PEG_2k_) with an aqueous mRNA phase using microfluidics, encapsulating antigen mRNA within lipid nanoparticles (LNP@mRNA). Nb‐PEG‐Cholesterol was subsequently attached to the LNP@mRNA surface, creating Nb‐LNP@mRNA. Following substantial uptake by DCs, the Nb‐LNP@mRNA platform efficiently delivered the encoded antigen mRNA, leading to its expression and subsequent processing into peptides within the DCs. Furthermore, engagement of the cell surface receptor PLS2 by the Nb‐LNP promoted DCs maturation and inhibited tumor cell proliferation, in part through the downregulation of the JAK2‐STAT3 signaling pathway. Mature DCs processed the antigen proteins into peptides and presented them on MHC molecules to T cells. This antigen presentation induced the differentiation of T cells into specialized effector cells, including CD4*^+^* helper T cells that regulate immune responses and antigen‐specific cytotoxic T lymphocytes that directly target and destroy tumor cells. This process thereby inhibits tumor growth via T cell‐mediated tumor killing and anti‐tumor cytokine secretion. The figure schematically depicts these steps, demonstrating the potential of our Nb‐LNP@mRNA vaccine approach for eliciting a potent anti‐tumor immune response in cancer immunotherapy.

In this study, we developed a DCs‐preferential mRNA delivery platform by decorating LNPs with the nanobody DC2.1 (Nb‐LNP@mRNA). We demonstrate that the Nb‐LNP platform achieves predominant DC targeting (up to 93%) mediated by an interaction between DC2.1 and the DC membrane protein Plastin‐2 (PLS2). This targeted delivery significantly enhanced intracellular mRNA expression. Furthermore, an Nb‐LNP@ LMP2 vaccine encoding the Epstein‐Barr virus (EBV) LMP2 antigen markedly inhibited EBV‐positive tumor growth in vivo, elicited potent anti‐tumor immunity, critically unleashing the full antigen‐presenting potential of DCs by downregulating the Leptin (Lep)‐JAK2‐STAT3 signaling pathway, a key negative regulator of DCs maturation. This study introduces a dual‐function Nb‐LNP platform, establishes PLS2 as a new targetable receptor for DCs delivery and immunomodulation, and presents a robust strategy for developing more effective mRNA cancer vaccines (Figure 1).

## 2 Results

### 2.1 Design and Characterization of a DC‐targeting nanobody‐LNP Platform

**FIGURE 2:**
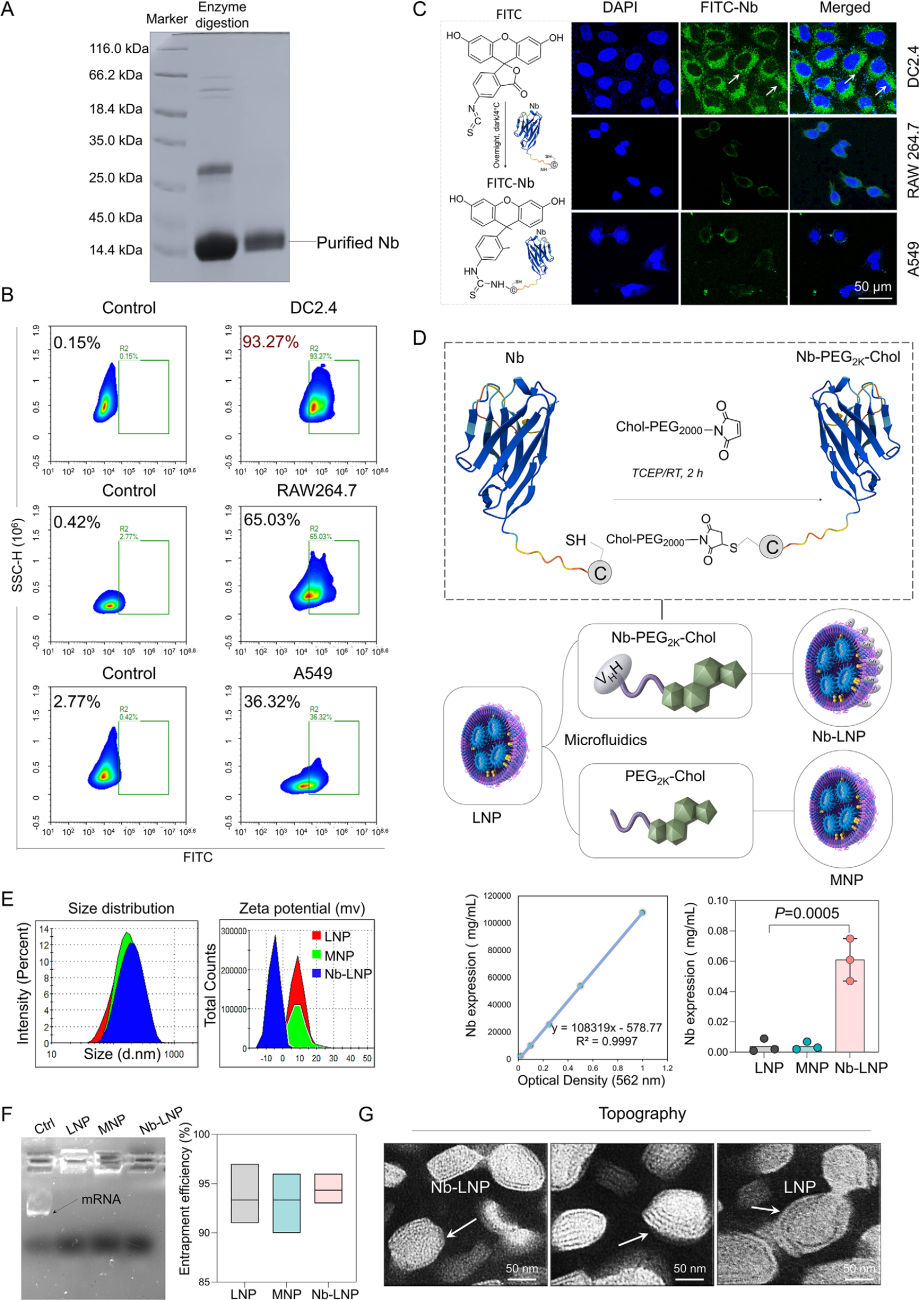
Design and characterization of the DC2.1 nanobody‐LNP platform. (A) Protein gel analysis of purified nanobody DC2.1 following enzyme digestion. The molecular weight marker is shown on the left. (B) Flow cytometry analysis demonstrating binding of nanobody DC2.1 to DC2.4, RAW264.7, and A549 cells. Control represents unstained cells. The percentage of cells in the gated region (R2) is indicated. (C) Immunofluorescence images of DC2.4, RAW264.7, and A549 cells incubated with FITC‐labeled nanobody DC2.1 (green) and stained with DAPI (blue) for nuclei visualization. Colocalization of green and blue signals appears as merged signals. White arrows indicate internalized nanobodies. Scale bar = 50 µm. (D) Schematic diagram illustrating the conjugation of nanobody DC2.1 to lipid nanoparticles (LNPs) via a PEGylated cholesterol linker (Nb‐PEG_2k_‐Chol) to create Nb‐LNPs. (E) Physicochemical characterization of LNP, MNP, and Nb‐LNP formulations. (Left) Size distribution and (middle) zeta potential measurements detected by DLS. (Right) Quantification of nanobody conjugation on LNPs using a standard curve generated from known concentrations of purified DC2.1 (inset). The bar graph shows nanobody expression (mg/mL) associated with LNP, MNP, and Nb‐LNP formulations. Data are presented as mean ± SEM (*n* = 3). p‐values were determined by one‐way ANOVA. (F) Agarose gel electrophoresis analysis of mRNA encapsulation within the LNP, MNP, and Nb‐LNP formulations (left), and quantification of entrapment efficiency (right). (G) Cryo‐transmission electron microscopy (TEM) images of Nb‐LNP and LNP, revealing nanoparticle morphology. White arrows highlight the particle structure.

Prior to formulating the nanobody‐modified LNPs (Nb‐LNPs), we rigorously characterized the nanobody‐PEGylated cholesterol conjugate (Chol‐PEG2K‐Ma‐Nb). SDS‐PAGE analysis provided direct evidence of successful conjugation, revealing a distinct molecular weight shift from approximately 15 kDa for the unconjugated nanobody to approximately 25 kDa for the conjugate, which perfectly aligned with the ∼10 kDa molecular weight of the Chol‐PEG‐MA moiety (Figure S1A). The conjugation process yielded a high coupling efficiency of approximately 92%, which remained highly stable over a 9‐day evaluation period (Figure S1B). Furthermore, binding kinetic assays confirmed that the conjugated nanobody fully retained its functional integrity, exhibiting a robust, high‐affinity interaction with the target PLS2 receptor (*K_d_* = 5.46 × 10^−9^ m) (Figure S1C). To develop a delivery system for specific DC‐targeting, we first characterized the binding properties of the nanobody DC2.1 [24, 25]. Western blot analysis confirmed the successful purification of DC2.1, with a molecular weight of approximately 15 kDa [26] (Figure 2A). We then assessed its binding affinity to various cell lines using flow cytometry. DC2.1 exhibited the highest binding to the DC2.4 murine dendritic cell line (93.27% positive). Consistent with the known expression of Plastin‐2 in hematopoietic lineages, we also observed moderate binding to RAW264.7 macrophages (65.03% positive), while lower binding was detected in A549 human lung epithelial cells (36.32% positive), indicating a preferential affinity for professional antigen‐presenting cells (Figure 2B). Immunofluorescence imaging further visualized this interaction, showing strong binding and internalization of FITC‐labeled DC2.1 by DC2.4 cells, confirming its potent and preferential targeting of DCs (Figure 2C).

Building on this, we engineered a DC‐targeting delivery platform by conjugating DC2.1 to lipid nanoparticles (LNPs) via a PEGylated cholesterol linker (Nb‐PEG_2k_‐Chol), creating Nb‐LNPs. Two control formulations were also prepared: unmodified LNPs and non‐targeting LNPs (MNP) (Figure 2D). Dynamic light scattering (DLS) measurements revealed that Nb‐LNPs possessed a uniform size distribution centered around 160 nm and a near‐neutral zeta potential of approximately −3 mV (Figure 2E, left and middle panels). To quantify the nanobody conjugation efficiency, we established a standard curve and confirmed a significantly higher amount of DC2.1 was present on the surface of Nb‐LNPs compared to the negligible background signals from control LNP and MNP formulations (p = 0.0005) (Figure 2E, right panel). Importantly, all three formulations (Nb‐LNP, LNP, and MNP) demonstrated high and comparable mRNA entrapment efficiencies (>90%), as assessed by agarose gel electrophoresis (Figure 2F). Finally, cryogenic transmission electron microscopy (cryo‐TEM) revealed the spherical morphology and lamellar structure of both Nb‐LNPs and LNPs (Figure 2G).

Collectively, these data demonstrate that the nanobody DC2.1 specifically targets DC2.4 cells and can be successfully incorporated into an LNP formulation, creating a well‐characterized Nb‐LNP platform with high mRNA loading capacity and structural integrity, poised for targeted delivery.

### 2.2 Nb‐LNP Specifically Targets DCs and Enhances mRNA Expression

**FIGURE 3:**
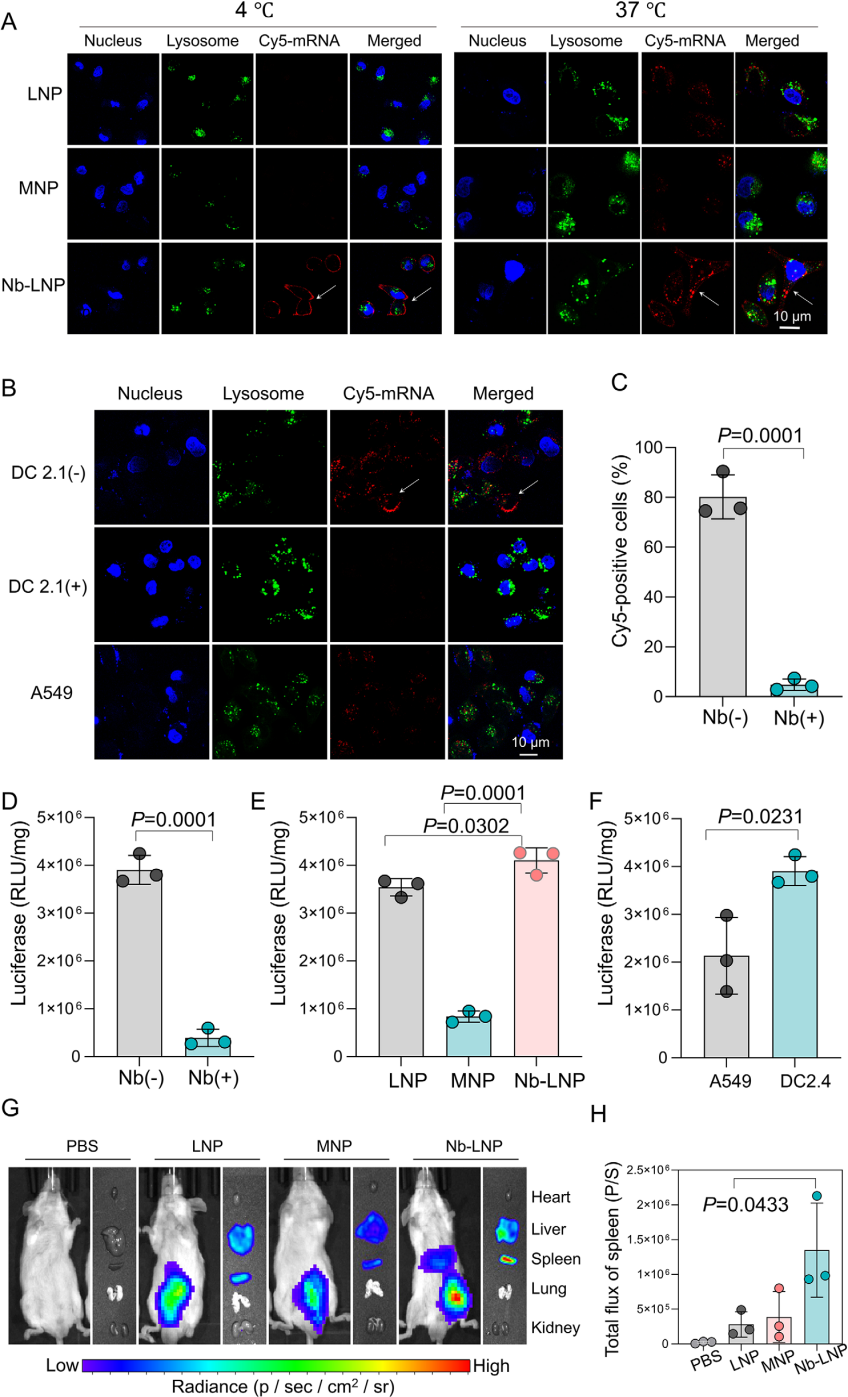
Detection of DC‐targeting by Nb‐LNP@mRNA. (A) Confocal microscopy images showing the binding (4°C) and uptake (37°C) of Cy5 mRNA delivered by LNP, MNP, or Nb‐LNP formulations in DC2.4 cells. Nuclei are stained with DAPI (blue), and lysosomes are stained with LysoTracker (green). Cy5 is shown in red. Note: Variations in lysosomal signal intensity (e.g., in the MNP group) reflect focal plane differences and field‐of‐view heterogeneity, rather than formulation‐induced biological effects. (B) Competitive inhibition assay in DC2.4 cells. Cells were pre‐incubated with (*^+^*) or without (‐) free DC2.1 nanobody before the addition of Nb‐LNP@Cy5. (C) Quantification of the binding capacity (at 4°C) of Nb‐LNP@mRNA in the competitive inhibition assay, measured as the percentage of Cy5‐positive cells. (D) Quantification of luciferase expression from Nb‐LNP@Fluc in a competitive inhibition assay, demonstrating that functional mRNA delivery is mediated by the DC2.1 nanobody. (E) Luciferase expression levels in DC2.4 cells 24 h after transfection with different FLuc formulations. (F) Comparison of luciferase expression levels from Nb‐LNP@FLuc in target DC2.4 cells vs. non‐target A549 cells. (G), (H) in vivo biodistribution and expression of Nb‐LNP@FLuc‐mRNA. (G) Representative IVIS images of whole mice and excised major organs (from top to bottom: heart, liver, spleen, lung, and kidneys) 6 h post‐subcutaneous injection at a dose of 20 µg FLuc‐mRNA per mouse. (H) Quantification of the total luciferase flux in the spleen. Data in (C‐F, H) are presented as mean ± SEM from biological replicates (*n* = 3 for C‐F; *n* = 3 for H). p‐values were determined by one‐way ANOVA or *t*‐test as appropriate. p‐values were determined by one‐way ANOVA or *t*‐test as appropriate.

Having established a well‐characterized platform, we next investigated its ability to specifically target and functionally deliver mRNA to DC2.4 cells. We first assessed nanoparticle uptake under two conditions: at 4°C, where only binding occurs, and at 37°C, which permits energy‐dependent internalization. Confocal microscopy revealed that at both temperatures, Nb‐LNP@Cy5 exhibited substantially stronger association with DC2.4 cells compared to the non‐targeting LNP@Cy5 and MNP@Cy5 controls (Figure 3A).

To rigorously confirm that this enhanced uptake was mediated by the DC2.1 nanobody, we performed a competitive inhibition assay. Pre‐incubation of DC2.4 cells with free DC2.1 nanobody dramatically reduced the subsequent binding and internalization of Nb‐LNP@Cy5 (Figure 3B–D). This result unequivocally demonstrates that the targeting of DCs by our platform is entirely dependent on the specific interaction of DC2.1 with its receptor.

We then evaluated whether this superior targeting translated into enhanced mRNA expression. Using luciferase‐encoding mRNA (Fluc mRNA), we found that the expression efficiency of Nb‐LNP@Fluc in DC2.4 cells was significantly higher than that of both LNP@Fluc and MNP@Fluc (Figure 3E). Furthermore, the specificity of the platform was highlighted by comparing its performance in target vs. non‐target cells; luciferase expression from Nb‐LNP@Fluc in DC2.4 cells (approximately 4.0 × 10^6^ RLU/mg) was approximately 2.2‐fold higher than in A549 cells (approximately 1.8 × 10^6^ RLU/mg) (Figure 3F). Finally, we validated these findings in vivo using an IVIS imaging system. Mice administered with Nb‐LNP@Fluc showed significantly higher luciferase signal in the spleen, a key secondary lymphoid organ rich in DCs, compared to all control groups (Figure 3G, H). To further delineate the in vivo cellular targeting profile upon vaccination, we evaluated the uptake of Cy5‐labeled nanoparticles across diverse immune cell subsets using flow cytometry (Figure S3). In tumor‐draining lymph nodes (dLNs), Nb‐LNP significantly enhanced uptake predominantly in total DCs. Furthermore, phenotypic assessment of these DCs revealed a significantly enhanced internalization by both the cross‐presenting cDC1 and cDC2 subsets. In the spleen, uptake was markedly elevated in DCs, as well as in macrophages and neutrophils, which aligns with the ubiquitous expression of the PLS2 target receptor on actively phagocytic leukocytes. While a moderate increase in uptake by T and B lymphocytes was noted in the spleen for the Nb‐LNP group, their absolute internalization levels remained substantially lower than those of the myeloid populations. Conversely, the LNP and MNP groups exhibited pronounced signals in the intestinal tract, a pattern characteristic of the canonical hepatic clearance and subsequent biliary excretion typical of unmodified nanoparticles. By modifying the nanoparticle surface with the DC2.1 nanobody, our platform effectively circumvented this non‐specific systemic clearance, actively redirecting the mRNA payload toward targeted splenic accumulation. These findings collectively demonstrate that the Nb‐LNP platform specifically targets DCs and substantially enhances functional mRNA delivery and expression, both in vitro and in vivo. To investigate the intracellular trafficking mechanisms, we evaluated the internalization pathways of Nb‐LNP in DC2.4 cells using specific endocytosis inhibitors (Figure S2A). The cellular uptake of Nb‐LNP was significantly reduced at 4°C and by chlorpromazine (CPZ), whereas filipin (Fil) and amiloride (Ami) showed no significant inhibitory effects. These results indicate that Nb‐LNP does not bypass the endosomal compartment, but is internalized via an energy‐dependent, clathrin‐mediated endocytosis pathway. Thus, the enhanced cytosolic mRNA delivery facilitated by the nanobody is primarily attributed to the increased intracellular accumulation of nanoparticles following PLS2 receptor engagement, rather than an alteration of the fundamental endosomal escape mechanism governed by the ionizable lipid IC2.

### 2.3 Nanobody Modification Alters the LNP Protein Corona

**FIGURE 4:**
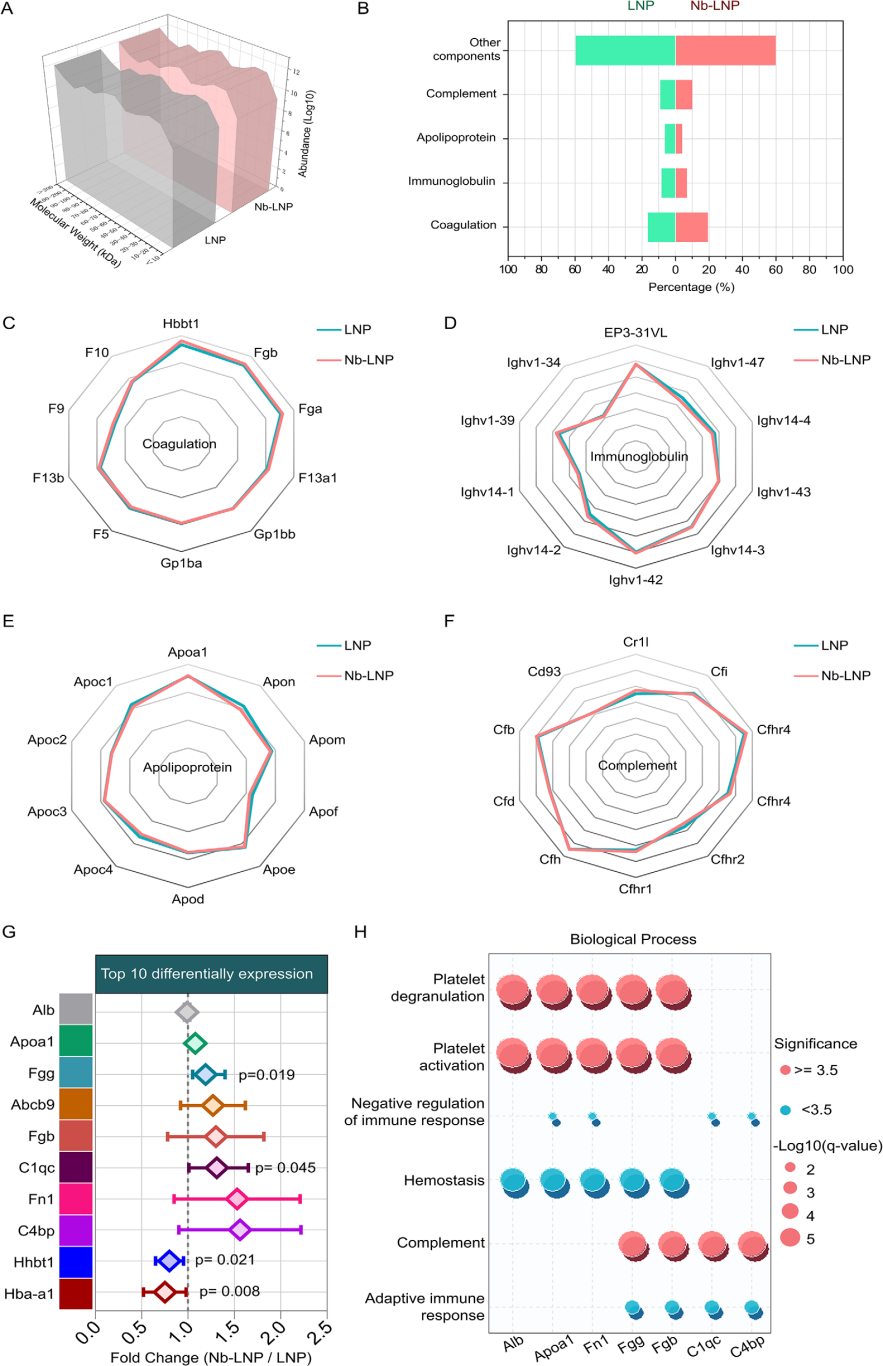
Proteomic characterization of LNP and Nb‐LNP protein coronas. (A) 3D distribution plot of protein abundance vs. molecular weight for the protein coronas formed on LNPs and Nb‐LNPs after plasma incubation. (B) Comparison of the relative abundance (%) of major protein families in the protein coronas of LNPs vs. Nb‐LNPs. (C–F) Radar plots comparing the relative abundance of representative proteins within specific functional classes: (C) coagulation factors, (D) immunoglobulins, (E) apolipoproteins, and (F) complement proteins. (G) Forest plot of the top 10 most differentially abundant proteins, showing fold change (Nb‐LNP / LNP) and p‐values. (H) Gene Ontology (GO) enrichment analysis of differentially abundant proteins. Bubble size corresponds to the statistical significance (‐Log10(q‐value)), and color represents the significance level.

To investigate the impact of nanobody modification on the biological characteristics of LNPs, we performed a proteomic analysis of the protein coronas formed on LNPs and Nb‐LNPs following incubation in plasma. The composition of the protein corona is a critical determinant of a nanoparticle's biological identity, profoundly influencing its in vivo circulation, cellular uptake, and targeting efficacy [27, 28, 29, 30]. Our analysis first revealed that both LNPs and Nb‐LNPs adsorbed a wide range of proteins from plasma, with similar overall protein abundance‐molecular weight distribution profiles (Figure 4A). However, a categorical comparison of the corona components highlighted key differences: compared to that of unmodified LNPs, the protein corona of Nb‐LNPs was significantly enriched in immunoglobulins, with a corresponding decrease in the relative abundance of coagulation factors and apolipoproteins (Figure 4B).

A detailed analysis of key protein classes revealed specific remodeling of the protein corona. Radar plots showed a consistent enrichment of various immunoglobulins on Nb‐LNPs (Figure 4D), while profiles for coagulation factors (Figure 4C), apolipoproteins (Figure 4E), and complement proteins (Figure 4F) exhibited more nuanced trends. To pinpoint statistically significant changes, we performed quantitative differential analysis (Figure 4G). This analysis identified a specific signature of differentially adsorbed proteins. Notably, the adsorption of fibrinogen gamma chain (Fgg) and complement C1q subcomponent (C1qc) was significantly enriched. Conversely, hemoglobin subunits (Hba‐a1, Hhbt1) were significantly reduced on the Nb‐LNP surface. This indicates that nanobody conjugation selectively reshapes the protein corona, rather than causing a global disruption. GO enrichment analysis of the differential proteins indicated an upregulation of pathways related to platelet activation and the complement system (Figure 4H). This suggests that the remodeled protein corona, through enhanced binding of fibrinogen and C1q, strengthens the nanoparticle's interaction with the immune system [31].

### 2.4 Nb‐LNP Targets DCs via PLS2 and Enhances Antigen Presentation by Inhibiting the Lep‐JAK2‐STAT3 Signaling Pathway

**FIGURE 5:**
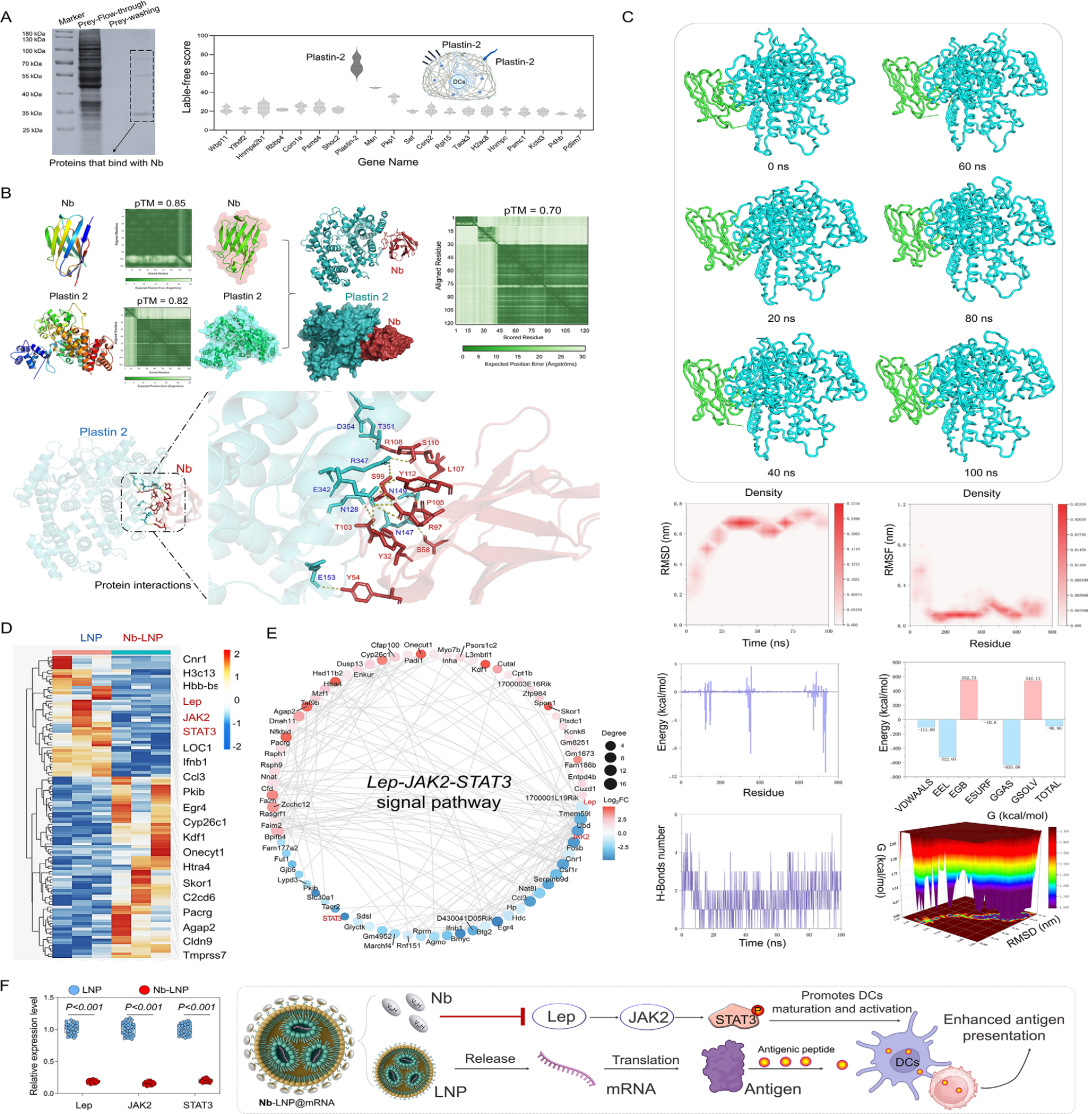
Nb‐LNP targets PLS2 on dendritic cells and modulates the Lep‐JAK2‐STAT3 signaling pathway to enhance immune response. (A) Identification of PLS2 as the binding partner for nanobody DC2.1. Left: SDS‐PAGE analysis of proteins pulled down by DC2.1‐conjugated beads. Right: Label‐free proteomics data showing Plastin‐2 (PLS2) as the most significantly enriched binding protein. (B) In silico modeling of the DC2.1‐PLS2 interaction. Top: AlphaFold‐predicted structures of DC2.1 (Nb, red) and PLS2 (cyan), and their docked complex, with corresponding model confidence plots (Predicted Template‐Modeling score, pTM). Bottom: A detailed view of the binding interface showing key interacting amino acid residues. (C) Molecular dynamics (MD) simulation confirms the stability of the DC2.1‐PLS2 complex. Top: Representative snapshots of the complex over a 100 ns simulation. Bottom: Analysis of simulation trajectories, including RMSD, RMSF, and binding free energy (MM/GBSA), all indicating a stable interaction. (D) Heatmap of differentially expressed genes in DCs treated with Nb‐LNP vs. control LNP, showing a distinct cluster of downregulated genes in the Nb‐LNP group. (E) Protein‐protein interaction (PPI) network analysis of the downregulated genes identified in (D). The analysis highlights the Lep‐JAK2‐STAT3 pathway as a key modulated signaling cascade. (F) Validation of pathway inhibition and schematic of the proposed mechanism. Left: Relative mRNA expression levels of *Lep*, *JAK2*, and *STAT3* are significantly decreased in DCs after Nb‐LNP treatment compared to the LNP control group. Data are presented as mean ± SD (*n* = 3). Statistical significance was determined by Student's *t*‐test. *p* < 0.001. Right: Schematic diagram illustrating how Nb‐LNP targets PLS2 on DCs and inhibits the Lep‐JAK2‐STAT3 pathway to promote DC maturation and enhance antigen presentation.

The nanobody DC2.1 was previously reported to target dendritic cells (DCs), but its specific ligand remained unclear [32, 33]. To identify this binding partner, we conducted a pull‐down assay using DC2.1, followed by label‐free quantitative proteomics. This analysis identified Plastin‐2 (PLS2), a known cytoskeletal protein expressed in hematopoietic cells [34], as the top candidate with the highest binding score, suggesting it is the primary high‐affinity partner for DC2.1 on the DC surface (Figure 5A).

To understand the molecular basis of this interaction, we predicted the 3D structures of both DC2.1 and PLS2 and modeled their protein‐protein complex using AlphaFold. The high confidence scores (pTM > 0.8) suggested a reliable prediction. The resulting model revealed a specific binding interface with multiple potential hydrogen bonds and hydrophobic interactions between the two proteins (Figure 5B). To assess the stability of this predicted complex, we performed a 100 ns molecular dynamics (MD) simulation. Analyses of Root Mean Square Deviation (RMSD), Root Mean Square Fluctuation (RMSF), and binding free energy (MM/GBSA) all confirmed that the DC2.1‐PLS2 complex is structurally stable and the binding is energetically favorable, strongly supporting a specific and stable interaction (Figure 5C). However, it is important to acknowledge that this 3D complex represents an in silico predictive model. While the computational data provide high‐confidence insights, future empirical structural characterizations utilizing high‐resolution techniques, such as X‐ray crystallography or cryogenic electron microscopy (cryo‐EM), are warranted to definitively resolve the exact atomic binding interface.

Having established that DC2.1 targets PLS2, we investigated the downstream immunological effects. We compared the transcriptomes of DCs treated with Nb‐LNP vs. control LNP. This analysis revealed that Nb‐LNP treatment led to the significant downregulation of a cluster of genes (Figure 5D). Protein‐protein interaction network analysis further showed that these downregulated genes were highly enriched in the Lep‐JAK2‐STAT3 signaling pathway (Figure 5E). To validate this key finding from our transcriptomic data, we measured the mRNA levels of the core pathway components. Quantitative RT‐PCR confirmed a significant decrease in the expression of Lep, JAK2, and STAT3 in DCs treated with Nb‐LNP (Figure 5F).

Collectively, these results uncover a complete mechanism: the Nb‐LNP system specifically targets DCs by binding to PLS2, which in turn inhibits the Lep‐JAK2‐STAT3 signaling cascade. The suppression of this pathway promotes DC maturation and activation, ultimately leading to enhanced antigen presentation (Figure 5F, Schematic).

### 2.5 Nb‐LNP@OVA Initiates Potent Anti‐tumor Response

**FIGURE 6:**
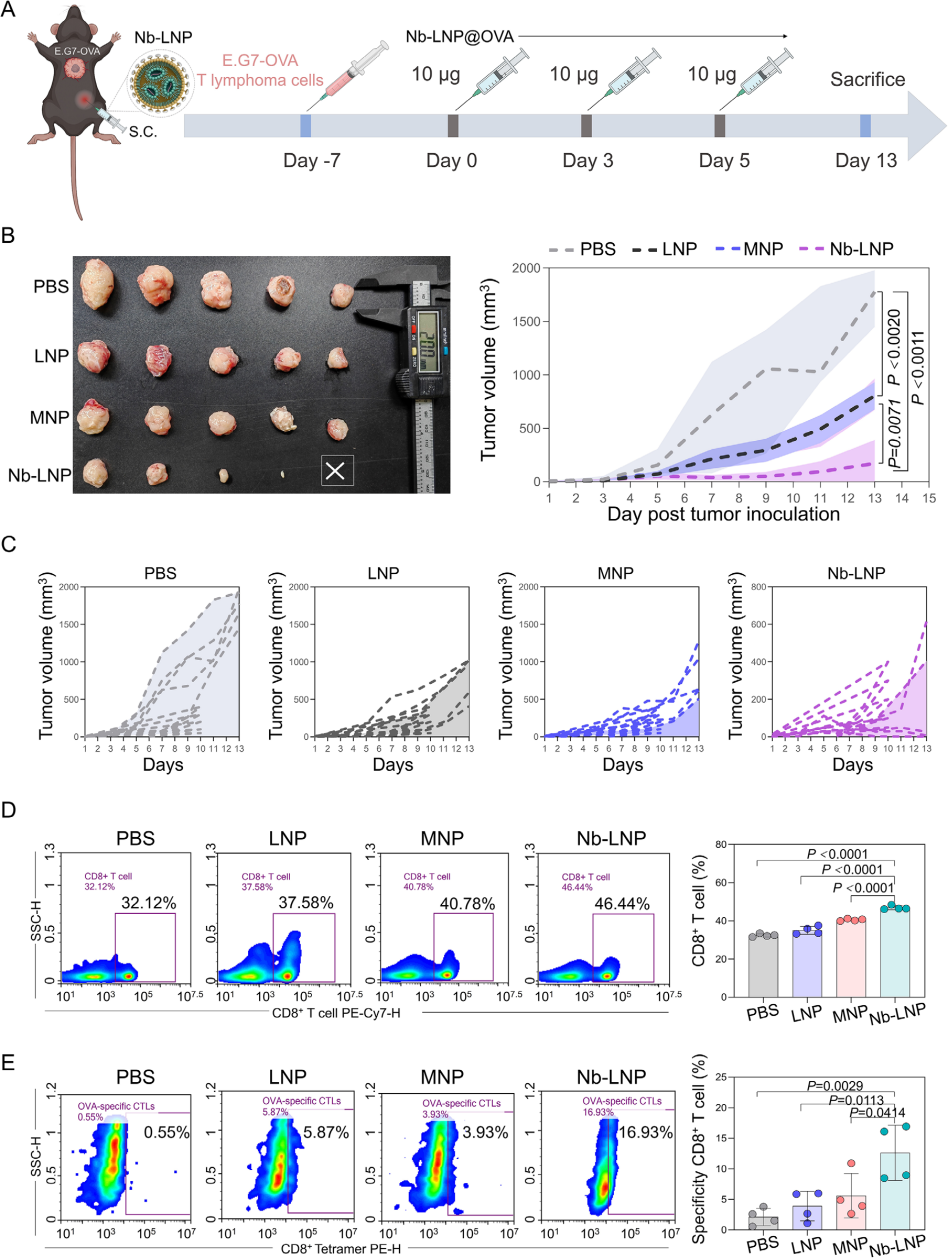
The Nb‐LNP‐delivered OVA mRNA vaccine suppresses tumor growth and elicits a robust antigen‐specific T cell response. (A) Schematic of the experimental timeline. C57BL/6 mice were subcutaneously inoculated with E.G7‐OVA cells on day ‐7. Mice received subcutaneous injections (S.C.) of PBS, LNP@OVA, MNP@OVA, or Nb‐LNP@OVA (10 µg mRNA) on days 0, 3, and 5. Analyses were performed on day 13. (B) Tumor burden analysis on day 13. Left: representative photographs of excised tumors from each group. Right: average tumor volume over time for each group. Data are presented as mean ± SD (*n* = 5). (C) Tumor growth trajectories for individual mice. (D) Flow cytometry analysis of T cell responses in tumor‐draining lymph nodes. Representative flow cytometry plots and quantification of the percentage of total CD8*^+^* T cells. Data are presented as mean ± SD (*n* = 4). (E) Representative flow cytometry plots and quantification of OVA‐specific CD8*^+^* T cells within the lymphocyte gate. Data are presented as mean ± SD (*n* = 4). Statistical significance was determined by one‐way ANOVA followed by Tukey's multiple comparisons test. p‐values are indicated on the graphs.

mRNA vaccines targeting dendritic cells significantly enhance antigen presentation efficiency and T‐cell immune responses, thereby increasing vaccine efficacy. This approach has demonstrated broad potential for applications in cancer immunotherapy and infectious disease prevention [35, 36, 37]. To evaluate the therapeutic efficacy of the Nb‐LNP delivery system, we developed an OVA‐encoding mRNA vaccine (Nb‐LNP@OVA) and tested it in an E.G7‐OVA syngeneic lymphoma model. The experiment followed the vaccination and analysis timeline depicted in Figure 6A, where tumor‐bearing mice received three intravenous doses of the formulations. Upon completion of the study on day 13, endpoint analysis of excised tumors revealed a profound anti‐tumor effect in the Nb‐LNP@OVA group. Representative images of the tumors visually confirmed this marked reduction in tumor burden, with some mice in the Nb‐LNP@OVA group showing nearly complete tumor regression (Figure 6B‐Left).

This superior therapeutic outcome was consistent with the tumor growth kinetics monitored throughout the experiment. While the LNP@OVA and MNP@OVA treatments demonstrated moderate tumor growth inhibition compared to the PBS control, the Nb‐LNP@OVA vaccine potently suppressed tumor growth (Figure 6B‐Right and Figure 6C). By day 13, the average tumor volume in the Nb‐LNP@OVA group was significantly smaller than that in the PBS (*p* < 0.0011) and MNP (p = 0.0071) groups, underscoring its superior ability to control tumor progression.

To investigate the immunological mechanism underlying this enhanced tumor control, we analyzed T cell populations in tumor‐draining lymph nodes by flow cytometry. The Nb‐LNP@OVA vaccine induced a significant expansion of the total CD8^+^ T cell population compared to all control groups (*p* < 0.0001 for all comparisons) (Figure 6D). More critically, the vaccination elicited a robust, antigen‐specific CD8^+^ T cell response. The percentage of OVA‐specific cytotoxic T lymphocytes, identified by tetramer staining, was dramatically elevated in the Nb‐LNP@OVA group, showing a significant increase over the PBS (p = 0.0029), LNP (p = 0.0113), and MNP (p = 0.0414) groups (Figure 6E). This response was approximately 3‐ to 4‐fold greater than that induced by the LNP and MNP formulations. Collectively, these data demonstrate that the Nb‐LNP delivery platform effectively generates a potent, antigen‐specific CTL response, leading to superior tumor control and therapeutic efficacy.

### 2.6 Potent Anti‐tumor Activity and Safety of the Nb‐LNP@LMP2 mRNA Vaccine

**FIGURE 7:**
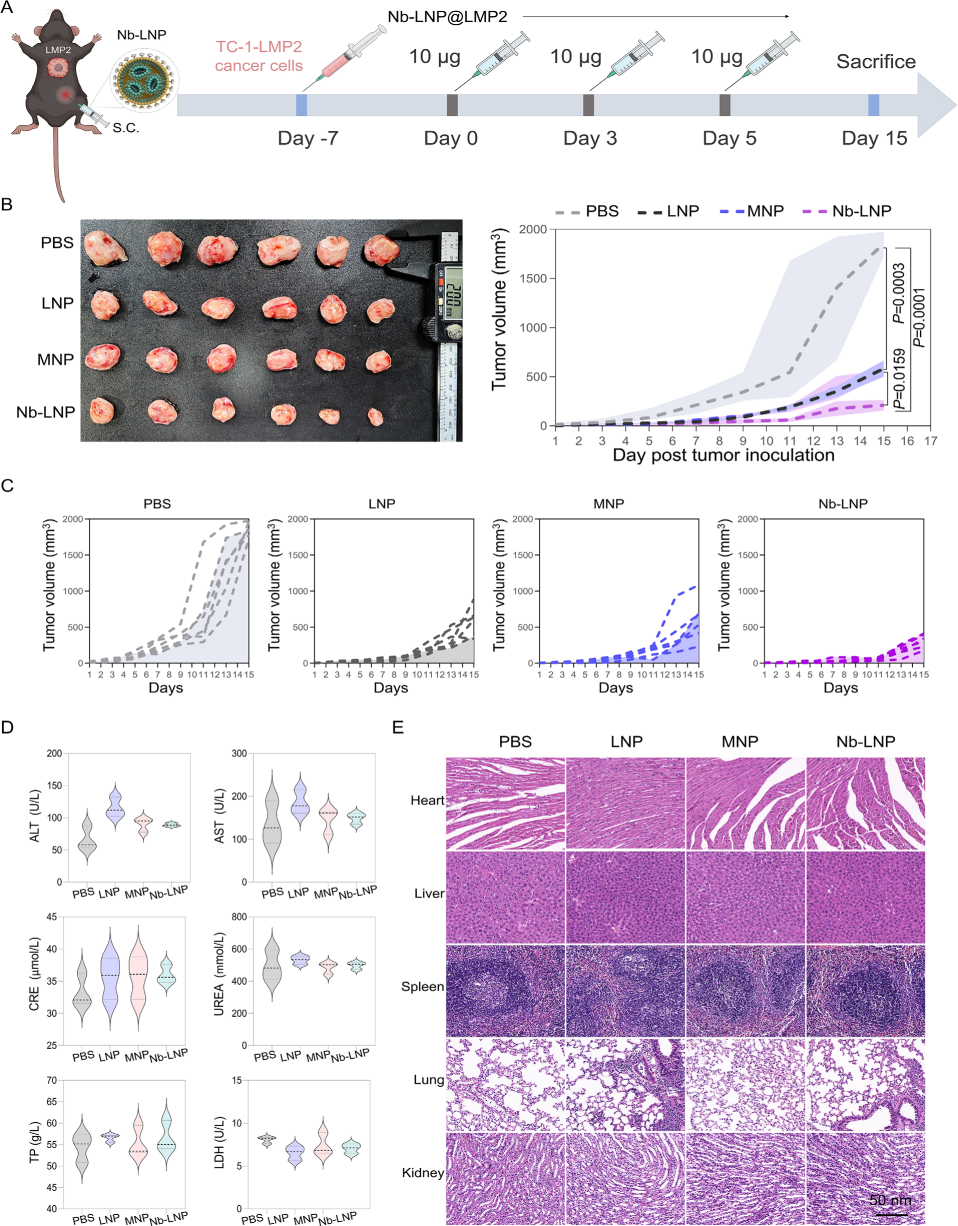
The Nb‐LNP@LMP2 mRNA vaccine inhibits tumor growth in vivo and shows a favorable safety profile. (A) Schematic of the therapeutic experiment. Tumor‐bearing mice were subcutaneous injected (S.C.) with LMP2 mRNA formulations (10 µ g/dose) on days 0, 3, and 5, with sacrifice and analysis on day 15. (B) Endpoint tumor analysis. Left: representative photographs of excised tumors from each group. Right: average tumor volume over time for each group. Data are presented as mean ± SD (*n* = 6). (C) Individual tumor growth trajectories within each group (mean ± SEM, *n* = 6). (D) Biosafety evaluation. Violin plots showing serum levels of key biochemical markers on day 15. (E) Representative H&E‐stained images of major organs from each treatment group. Scale bar = 50 µm. Statistical significance for (B) and (C) was determined by one‐way ANOVA with Tukey's multiple comparisons test. p‐values are indicated on the graphs.

To assess the therapeutic potential of our delivery system against a clinically relevant target, we developed an mRNA vaccine encoding the nasopharyngeal carcinoma (NPC)‐associated antigen LMP2. The in vivo anti‐tumor efficacy and safety of the Nb‐LNP@LMP2 vaccine were evaluated in a tumor‐bearing mouse model, following the experimental timeline depicted in Figure 7A.

At the study endpoint on day 15, a significant therapeutic benefit was observed in the group receiving the Nb‐LNP@LMP2. As shown in Figure 7B‐Left, mice treated with Nb‐LNP@LMP2 had a visibly smaller tumor burden compared to all control groups.

The superior efficacy of the Nb‐LNP@LMP2 vaccine was further supported by the tumor growth kinetics throughout the study. The tumor volume curves in Figure 7B ‐Right and Figure 7C illustrate that the Nb‐LNP formulation led to potent and sustained inhibition of tumor growth. Consequently, at the study endpoint on day 15, the final average tumor volume in the Nb‐LNP@LMP2 group was significantly smaller than that in the control groups (p = 0.0001 vs. PBS and p = 0.0159 vs. MNP).

In parallel with efficacy, we evaluated the systemic safety of the vaccine. The biosafety profile, assessed through serum biochemistry and organ histology, is presented in Figure 7D. Evaluation of key serum biochemical markers‐including indicators for liver function (alanine aminotransferase, ALT; aspartate aminotransferase, AST), kidney function (creatinine, CRE; urea), and general tissue health (total protein, TP; lactate dehydrogenase, LDH)‐revealed no significant differences among the treatment groups, indicating a favorable safety profile with no apparent hepatotoxicity or nephrotoxicity. Furthermore, histological examination of H&E‐stained sections from the heart, liver, spleen, lung, and kidney showed no signs of pathological abnormalities, inflammation, or tissue damage in any group (Figure 7E). Collectively, these results demonstrate that the Nb‐LNP@LMP2 vaccine is not only highly effective at suppressing tumor growth in vivo but also exhibits a favorable safety profile.

## 3 Discussion

Our study demonstrates that a nanobody‐LNP platform enables highly specific and efficient in vivo delivery of mRNA to DCs by targeting a previously unexploited surface receptor, PLS2. This approach not only dramatically enhances mRNA vaccine potency but also beneficially remodels the tumor immune microenvironment, offering a dual mechanism of anti‐tumor activity.

A central challenge in cancer immunotherapy is solving the puzzle of targeted delivery [6, 38]. Our work addresses this by designing a robust and modular nanobody‐LNP platform, whose straightforward construction and characterization underscore its potential for scalable and cost‐effective manufacturing compared to traditional antibody‐based approaches (Figures 1 and 2). The true power of this platform, however, lies in its dual functionality, which is entirely driven by the preferential interaction with its target. Our platform achieves superior DC uptake and dramatically higher antigen expression in vivo by targeting PLS2 [39, 40, 41] with the DC2.1 nanobody (Figure 3). Although PLS2 is also expressed in other leukocytes such as macrophages **(as demonstrated in** Figure 2B; Figure S3), this preferential binding likely contributed to the synergistic anti‐tumor response via supplementary antigen presentation. Mechanistically, we hypothesize that this pathway downregulation may be related to the structural perturbation of the PLS2 cytoskeletal scaffold at the membrane cortex [42]. Nanobody engagement likely induces localized actin remodeling or steric hindrance, potentially disrupting the Leptin receptor (LepR) signaling complex and uncoupling JAK2 from its receptor [43]. This relieves the immunosuppressive brake, facilitating DC maturation [44]. Furthermore, while the remodeled protein corona (enriched with immunoglobulins and C1q, Figure 4) provides a synergistic ‘immune‐priming’ microenvironment, the specific Nb‐PLS2 interaction remains the predominant driver of these effects. This is critically evidenced by the nearly complete abrogation of internalization upon PLS2 blockade (Figure 3B) and the precise, receptor‐specific downregulation of the Lep‐JAK2‐STAT3 cascade (Figure 5), outcomes unattainable through non‐specific corona‐mediated phagocytosis. Importantly, because PLS2 regulates actin‐bundling, nanobody engagement could theoretically impact DC motility. Based on our AlphaFold model (Figure 5B), the DC2.1 nanobody docks at a surface‐exposed region of PLS2. We hypothesize that this binding interface might be spatially distinct from the primary domains required for actin cross‐linking, potentially minimizing direct steric hindrance. Crucially, this structural speculation is strongly corroborated by our in vivo functional data. The robust expansion of antigen‐specific CD8^+^ T cells observed in tumor‐draining lymph nodes (Figure 6D) necessitates the active and efficient migration of mature DCs from the subcutaneous injection site. This outcome inherently demonstrates that Nb‐LNP targeting does not critically impair DC cytoskeletal dynamics or their migratory capacity in vivo.

It is this synergistic, dual mechanism‐precise targeting for enhanced antigen expression coupled with direct immunological relicensing DCs ‐that explains the platform's profound therapeutic efficacy. This combination drove a 3–4 fold increase in antigen‐specific CD8*^+^* T cells (Figure 6) and led to near‐complete tumor regression in aggressive tumor models, a result unattainable by non‐targeted LNPs (Figures 6 and 7). Our study thus demonstrates that a precisely engineered strategy, where targeting and relicensing DCs are intrinsically linked, can be the deciding factor between marginal efficacy and a curative response. These findings open up several exciting future avenues. A critical next step is to dissect the precise signaling cascade linking PLS2 engagement to JAK2‐STAT3 modulation, which could enable the design of next‐generation nanobodies with fine‐tuned immunomodulatory functions. Furthermore, investigating the expression profile of PLS2 across different DC subsets (e.g., cDC1 vs. cDC2) will be crucial for understanding its role in orchestrating distinct types of T cell responses and for tailoring vaccine strategies [45].

In summary, our nanobody‐LNP platform provides a powerful solution to the long‐standing challenge of DC‐specific targeting. By integrating highly efficient delivery with favorable immunomodulation, this work lays a robust foundation for a new generation of mRNA therapeutics designed to precisely orchestrate the immune system against cancer and other challenging diseases.

## 4 Materials and Methods

### 4.1 Materials

Ionizable lipid IC2 [46, 47], DOPE (cat#S03005), Cholesterol (cat#001001), and DMG‐PEG_2k_ (cat#O02005) were provided by AVT (Shanghai) Pharmaceutical Tech Co., Ltd (Shanghai, China), Low melting point agarose (UltraPure Low Melting Point Agarose; Invitrogen, cat#16520100) was purchased from Thermo Fisher Scientific (Waltham, MA, USA). ACK Lysing Buffer (Gibco, cat#A1049201) was purchased from Thermo Fisher Scientific (Waltham, MA, USA). APC‐conjugated anti‐mouse CD8a and PE‐conjugated anti‐mouse CD4 antibodies were purchased from BioLegend (cat#100712 for CD8α, cat#100406 for CD4, San Diego, CA, USA). PE anti‐mouse SIINFEKL/H‐2Kb OVA tetramer was obtained from BioLegend (cat#141603, San Diego, CA, USA). DMEM (cat#11965092), RPMI‐1640 medium (cat#11875085), fetal bovine serum (FBS) (cat#A5670701), and phosphate buffer (PBS) were purchased from Gibco (cat#20012050, Grand Island, NY, USA). Cryogenic refrigerated centrifuges (Cryofuge 8) were from Thermo Scientific (cat#75008603, Waltham, MA, USA). GFP mRNA, Luc mRNA, OVA mRNA, and LMP2 mRNA were synthesized in‐house according to established protocols [46, 47]. All other reagents and chemicals were analytical grade. His Protein Interaction Pull‐Down kit (Pierce) was purchased from Thermo Scientific (cat#21277, Waltham, MA, USA); Coomassie brilliant blue R‐250 was purchased from Sangon Biotech (cat#A610037, Shanghai, China).

### 4.2 Methods

#### 4.2.1 Ethics Statement

This study was carried out in accordance with the recommendations in the Guide for the Care and Use of Laboratory Animals of the National Institutes of Health. The protocols were approved by the Institutional Animal Care and Use Committee at Sichuan University. All the animal experimental procedures were performed under anesthesia that was induced and maintained with isoflurane, and all efforts were made to minimize animal suffering.

#### 4.2.2 Animals

C57BL/6 mice (6–8 weeks) were purchased from Beijing Huafukang Biotechnology Co., Ltd (China, Beijing). Mice were maintained in the animal facility at Sichuan University for two weeks before the experiment. Male animals were used with random grouping. All animal studies were approved by the Sichuan University Institutional Animal Care and Use Committee and performed following the animal care and institutional guidelines.

#### 4.2.3 Nanobody DC2.1 Preparation

The pET 22b recombinant plasmid containing the nanobody DC2.1 fusion protein gene was constructed and transformed into *E. coli* strains. The fusion protein was expressed and purified using Ni‐NTA resin by affinity chromatography (as shown in Figure 2A).

#### 4.2.4 Targeting DCs with nanobody DC2.1

To investigate the targeting ability of nanobody DC2.1 to DCs, we coupled DC2.1 with FITC fluorescent to obtain DC2.1‐FITC. DC2.1‐FITC was added to cell suspensions of A549, RAW264.7, and DC2.4, respectively, and incubated at 4°C for 0.5 h. The proportion of FITC‐positive cells was detected using flow cytometry. In addition, the uptake ability of the three types of cells for DC2.1‐FITC was detected using laser confocal microscopy.

#### 4.2.5 Preparation and Characterization of Nb‐LNP

DC2.1‐PEG_2k_‐Chol was prepared by mixing nanobody DC2.1 and TCEP in a molar ratio of 1:5 and reacting at room temperature for 2 h. TCEP was removed using a desalting column (HiTrap Desalting, 5 mL; cat#GE29‐0486‐84, Cytiva/GE Healthcare, Marlborough, MA, USA). The product was quickly mixed with Chol‐PEG_2k_‐Mal at room temperature for 4 h. Finally, excess Chol‐PEG_2k_‐Mal was removed using a 10 kDa ultrafiltration membrane, and the product was quantified using the BCA protein quantification method to obtain DC2.1‐PEG_2k_ ‐Chol.

#### 4.2.6 Determination of Nanobody Coupling Efficiency and Stability

To determine the coupling efficiency of the anti‐DC2.1 nanobody, the synthesized Chol‐PEG2k‐Mal‐Nb conjugate mixture (prior to ultrafiltration) was placed in a 100 kDa molecular weight cut‐off (MWCO) ultrafiltration tube and centrifuged at 2400 × g for 15 min. The filtrate, containing the unbound nanobody (*W_free_*), was collected and analyzed using a BCA protein assay kit (Beyotime, Shanghai, China). *W_total_* represented the total amount of anti‐DC2.1 nanobody added during the preparation. The coupling efficiency was calculated using the following equation: Coupling efficiency (%) = (*W_total_*‐*W_free_*) / *W_total_* × 100%. The storage stability of the conjugate was assessed by storing the formulation at 4°C for 3, 6, and 9 days, and subsequently measuring the free nanobody content in the filtrate to determine the remaining coupling efficiency over time.

#### 4.2.7 Preparation of LNP

The IC2, DCPE, Chol, and DMG‐PEG_2k_ were dissolved in anhydrous ethanol at the molar ratio of 35:16:46.5:2.5 to obtain the ethanol phase. The mRNA was diluted to a concentration of 0.33 mg/mL using a 10 mm pH = 6.0 citrate‐sodium citrate buffer to obtain the water phase. LNP was prepared using microfluidics at the volume ratio at 1:3 (The ethanol phase to the water phase).

#### 4.2.8 Preparation of MNP

MNP, serving as a non‐targeting control nanoparticle, was prepared using the same method as the standard LNP described above. This formulation contains the standard DMG‐PEG_2k_ and does not include a targeting nanobody, acting as a control for non‐targeted delivery.

#### 4.2.9 Preparation of Nb‐LNP

Nb‐LNP was prepared using the same lipid composition and microfluidic method as the LNP, but with the standard PEG‐lipid (DMG‐PEG_2k_) being replaced by an equimolar amount of the prepared DC2.1‐PEG_2k_‐Chol conjugate.

#### 4.2.10 Liposome Characterization

Zeta‐potential and average size of Nb‐LNP were measured by using Malvern Laser Particle Size Analyzer (Zetasizer Nano ZS 90, Malvern, UK). Data were obtained as an average of more than 3 measurements on different samples.

#### 4.2.11 Gel Retardation Assay

The mRNA‐loading ability of the different formulations was evaluated by gel retardation assay. Briefly, the gel was run at a constant voltage of 130 V for 20 min at room temperature, and was observed in a gel documentation system (Gel Doc 2000, Bio‐Rad Laboratories, Hercules, USA).

#### 4.2.12 mRNA Integrity

mRNA integrity was evaluated using a Bioptic RNA Cartridge Kit (Bioptic Inc., Taiwan, China).

#### 4.2.13 Transmission Electron Microscopy (TEM)

The morphology of nanoparticles was observed by TEM.

#### 4.2.14 Nb‐LNP Uptake by DCs

Cells (2 × 10^6^) were cultured overnight in 24‐well plates containing slides. Fresh medium containing 0.8 µg of Cy5‐LNP, Cy5‐MNP, and Cy5‐Nb‐LNP was added into cells, incubated in a humidified incubator at 4°C and 37°C with 5% CO_2_ for 1 h, respectively. 10 µL LysoTracker was added to each well, incubated for 15 min at room temperature, and washed with PBS three times for 3 min each time. Cells were fixed with 1 mL paraformaldehyde, and DAPI (Sigma–Aldrich) was used to stain the nucleus for 15 min in the dark at room temperature. The images were captured with a Zeiss Meta 510 confocal microscope (Carl Zeiss, Oberkochen, Germany). Cy5 mRNA was colored as red, the lysosome was colored as green, and the cell nucleus was colored as blue.

#### 4.2.15 In vivo Distribution of Nb‐LNP

Fluc‐Nb‐LNP was prepared by using Fluc mRNA (Fluc‐LNP and Fluc‐MNP as controls). Balb/c male mice (6–8 weeks old) received a subcutaneous injection of LNP@Fluc‐mRNA, MNP@Fluc‐mRNA, or Nb‐LNP@Fluc, each containing 20 µg of Firefly luciferase (Fluc)‐encoding mRNA. The mice were intraperitoneally injected with D‐luciferin potassium salt (150 mg/kg) at 6 h after administration and were anesthetized with isoflurane after 10 min. The fluorescence intensity was measured by an animal in vivo imaging system (IVIS Lumina system, Perkin–Elmer).

#### 4.2.16 Molecular Mechanism of Nb‐LNP Anti‐tumor

DC2.4 cells were cultivated to 80% fusion (2 × 10^6^ cells/well), and were transfected with 20 µg mRNA Nb‐LNP@OVA, incubated at 37°C for 24 h with 5% CO_2_ for 4 h, and washed three times with 1 mL PBS. Total RNA was extracted and sequenced by RNA sequencing.

#### 4.2.17 Identifying the Targeting Receptor

The binding receptor for nanobody DC2.1 was identified using a pull‐down assay coupled with label‐free quantitative proteomics. Briefly, His‐tagged DC2.1 protein was immobilized onto magnetic beads and subsequently incubated with whole‐cell lysate prepared from DC2.4 cells to capture binding partners. After extensive washing to remove non‐specific proteins, high‐affinity binders were eluted. The eluate was resolved by SDS‐PAGE, and protein bands specific to the DC2.1 bait were subjected to mass spectrometry for identification. The interaction between DC2.1 and its identified top binding partner, Plastin‐2 (PLS2), was then modeled in silico using AlphaFold 2 to predict the structure of their complex.

#### 4.2.18 Analysis of Downstream Signaling

##### 4.2.18.1 Transcriptome Analysis

To investigate the molecular mechanisms following receptor engagement, DC2.4 cells were treated with either control LNP or Nb‐LNP for 24 h. Total RNA was extracted (TRIzol Reagent), and high‐quality samples (RIN > 9.0) underwent library preparation (NEBNext Ultra II RNA Library Prep Kit) and were sequenced on an Illumina NovaSeq platform. Raw reads were aligned to the mouse genome (GRCm39), and differential expression analysis was performed using the DESeq2 package. Genes with an adjusted p‐value < 0.05 and |log2(Fold Change)| > 1 were considered differentially expressed. A heatmap was generated to visualize these genes (Figure 5D), and a protein‐protein interaction (PPI) network of the downregulated genes was constructed using the STRING database and Cytoscape to identify key modulated pathways (Figure 5E).

##### 4.2.18.2 qRT‐PCR Validation

Key findings from the RNA‐seq data were validated by quantitative real‐time PCR. cDNA was synthesized from RNA extracted from similarly treated cells. The relative mRNA expression levels of *Lep*, *JAK2*, and *STAT3* were quantified using the 2^−ΔΔCt^ method, with Gapdh serving as the housekeeping gene for normalization (Figure 5F).

#### 4.2.19 The Anti‐tumor Efficacy of Nb‐LNP based mRNA Vaccines

##### 4.2.19.1 E.G7‐OVA Lymphoma Model

C57BL/6 male mice (6–8 weeks old, *n* = 6 per group) were subcutaneously inoculated in the right flank with 3 × 10^5^ E.G7‐OVA cells in 100 µL of PBS. Tumor inoculation was performed on day ‐7, seven days prior to the first treatment. Following the timeline in Figure 6A, mice received subcutaneous injections of the respective vaccine formulations on days 0, 3, and 5. Tumor volume was monitored throughout the experiment.

##### 4.2.19.2 TC‐1‐LMP2 Tumor Model

Similarly, C57BL/6 male mice (6–8 weeks old, *n* = 6 per group) were subcutaneously inoculated with 3 × 10^5^ TC‐1‐LMP2 cells. The immunization schedule was the same as described for the lymphoma model.

#### 4.2.20 Detection of CD8*^+^* T Cells in Lymph Nodes and Tumor‐specific CTL in Tumor

The lymph nodes of the E.G7‐OVA tumor mice treated with Nb‐LNP@OVA were collected and filtered with a 45 µm sterile nylon filter, and the red blood cells in the lymph node cells were removed by using cell lysate to obtain a lymphocyte cell suspension. Similarly, the tumor tissue was cut into pieces, and was digested with a mixed solution of 0.5 mg/mL type I collagenase and 1 mg/mL type IV collagenase in a water bath at 37°C for 2 h, and was filtered with a 70 µm sterile nylon filter to obtain a tumor cell suspension. Add 0.1 µg of APC‐Cy7 anti‐mouse CD45, FITC anti‐mouse CD3, APC anti‐mouse CD4, PE‐Cy7 anti‐mouse CD8α, and PE anti‐mouse SIINFEKL/H‐2Kb OVA tetramer to the lymphocyte suspension, incubated at 4°C in the dark for 40 min, washed twice with sterile PBS buffer, and specific CD8*^+^* T cells and antigen‐specific T cells in lymph nodes and tumors were detected by flow cytometry.

#### 4.2.21 Statistical Analysis

All experiments were performed at least three times unless stated otherwise. Data were presented as mean ± SD or mean ± SEM. All data were analyzed with GraphPad Prism 10.0 software using One‐Way ANOVA plus Tukey's post‐hoc test; Statistically significant differences are indicated as ^*^p<0.05, ^**^p<0.01, ^***^p<0.001, ^****^p<0.0001.

## Author Contributions

S.Q., Z.H., H.H., Y.Z., and X.S. conceived and designed the study. S.Q., Z.H., H.H., and Y.Z. performed the majority of the experiments and analyzed the data. Y.C., X.H., Z.N., Z.X., and C.Z. assisted with the experiments. S.Q., Z.H., and H.H. wrote the initial manuscript. X.S. supervised the study, acquired funding, and revised the manuscript. All authors reviewed and approved the final manuscript.

## Conflicts of Interest

The authors declare no conflicts of interest.

## Supporting information




**Supporting File**: advs75062‐sup‐0001‐FigureS1–S3.pptx.

## Data Availability

The data that support the findings of this study are available from the corresponding author upon reasonable request.
