# Supplementary material for: A Nanobody‐LNP Platform for Targeting and Relicensing Dendritic Cells for Potent Cancer Immunotherapy
Source: Adv Sci (Weinh). 2026 May 4;13(34):e23024. doi: 10.1002/advs.202523024 (PMC13285146; doi:10.1002/advs.202523024)
Supplement: Supplementary file 1 — Supporting File: advs75062‐sup‐0001‐FigureS1–S3.pptx. [file ADVS-13-e23024-s001.pptx]

## Slide 1
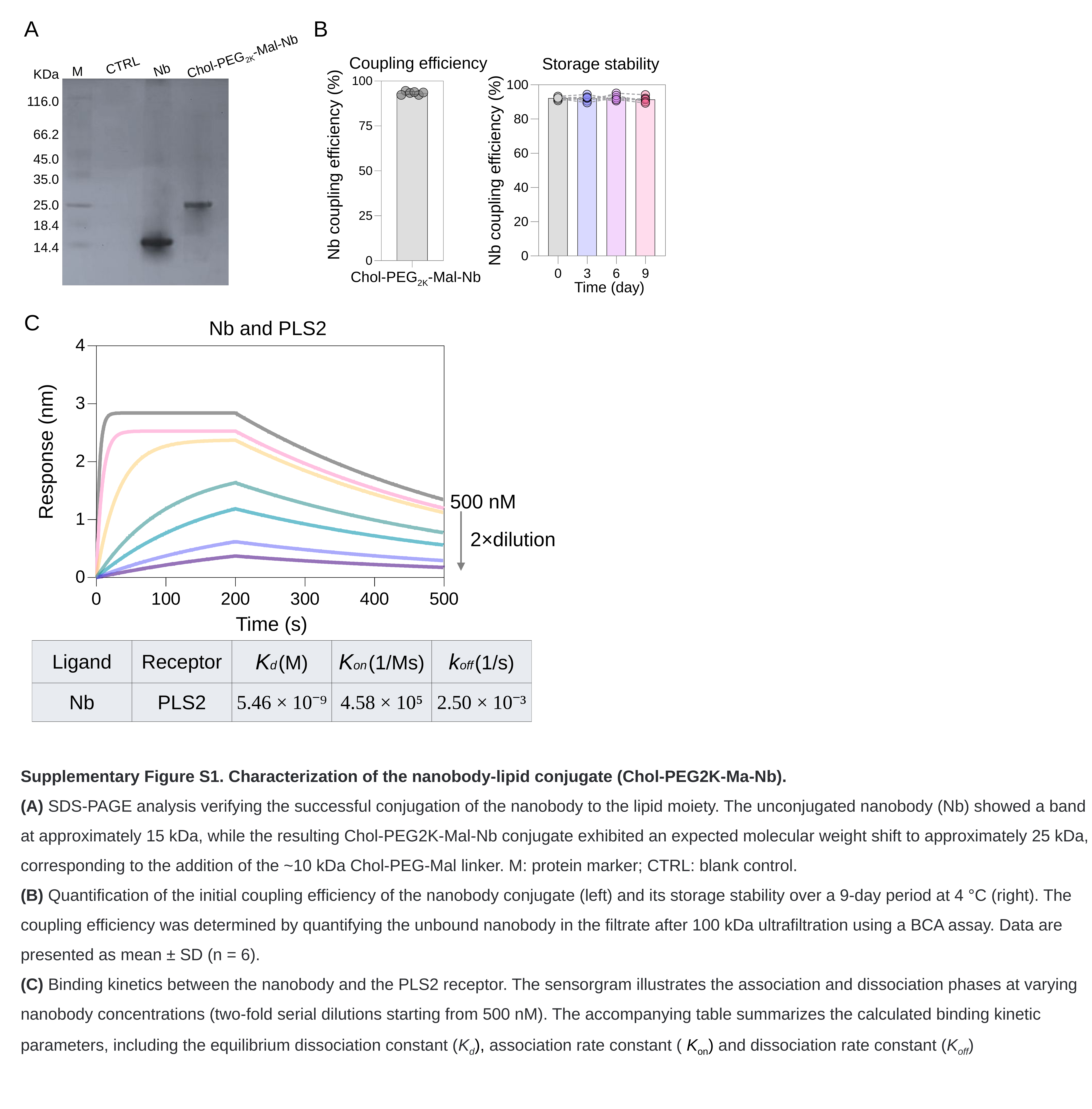

A
B
Chol-PEG2K-Mal-Nb
CTRL
Nb
M
KDa
116.0
66.2
45.0
35.0
25.0
18.4
14.4
Coupling efficiency
Storage stability
Nb coupling efficiency (%)
Nb coupling efficiency (%)
Chol-PEG2K-Mal-Nb
Time (day)
C
Nb and PLS2
Response (nm)
500 nM
2×dilution
Time (s)
| Ligand | Receptor | Kd​ (M) | Kon​ (1/Ms) | koff​ (1/s) |
| --- | --- | --- | --- | --- |
| Nb | PLS2 | 5.46 × 10⁻⁹ | 4.58 × 10⁵ | 2.50 × 10⁻³ |
Supplementary Figure S1. Characterization of the nanobody-lipid conjugate (Chol-PEG2K-Ma-Nb).
(A) SDS-PAGE analysis verifying the successful conjugation of the nanobody to the lipid moiety. The unconjugated nanobody (Nb) showed a band at approximately 15 kDa, while the resulting Chol-PEG2K-Mal-Nb conjugate exhibited an expected molecular weight shift to approximately 25 kDa, corresponding to the addition of the ~10 kDa Chol-PEG-Mal linker. M: protein marker; CTRL: blank control.
(B) Quantification of the initial coupling efficiency of the nanobody conjugate (left) and its storage stability over a 9-day period at 4 °C (right). The coupling efficiency was determined by quantifying the unbound nanobody in the filtrate after 100 kDa ultrafiltration using a BCA assay. Data are presented as mean ± SD (n = 6).
(C) Binding kinetics between the nanobody and the PLS2 receptor. The sensorgram illustrates the association and dissociation phases at varying nanobody concentrations (two-fold serial dilutions starting from 500 nM). The accompanying table summarizes the calculated binding kinetic parameters, including the equilibrium dissociation constant (Kd), association rate constant ( Kon) and dissociation rate constant (Koff)

## Slide 2
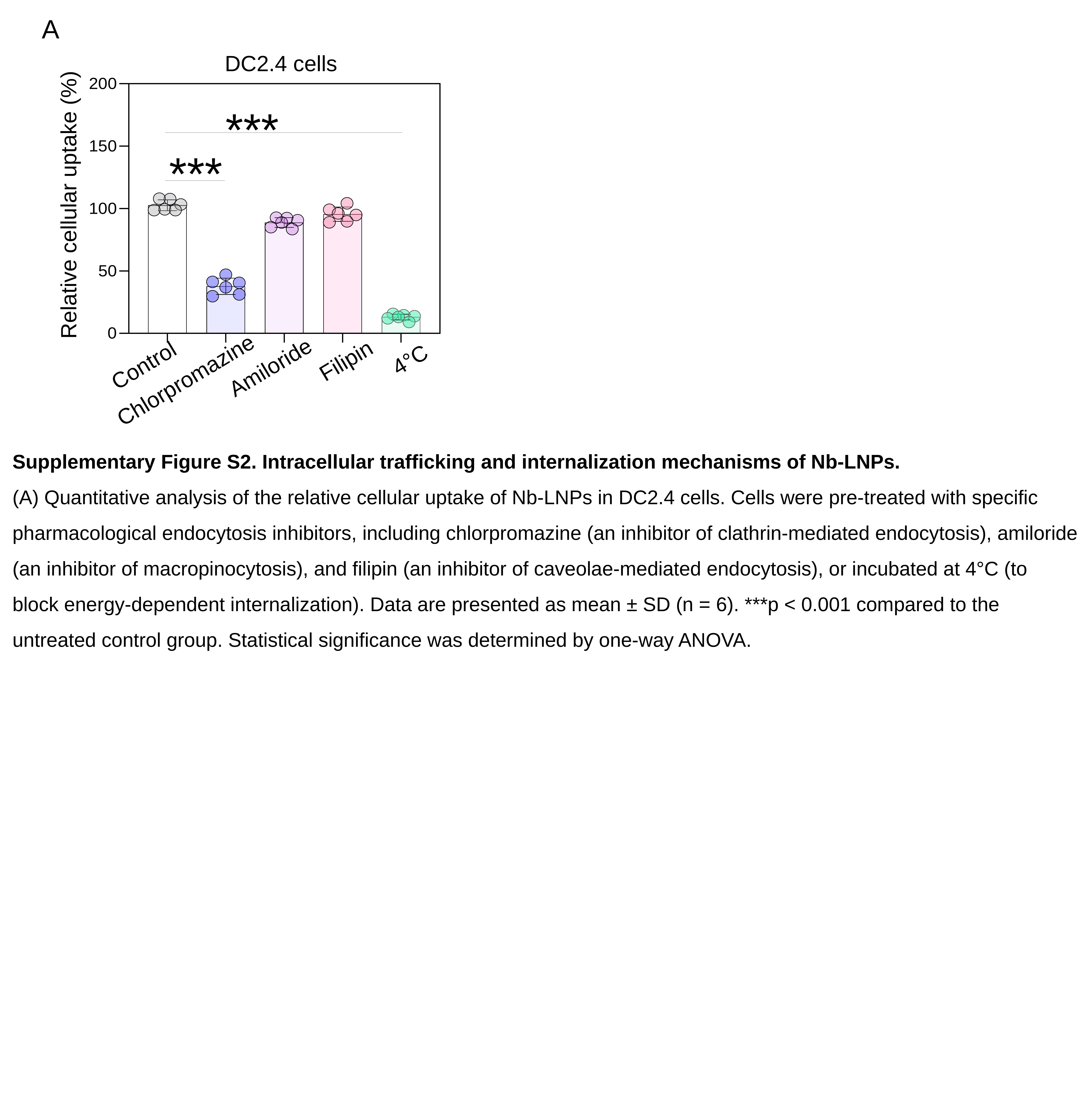

A
DC2.4 cells
Relative cellular uptake (%)
4°C
Control
	Filipin
	Amiloride
Chlorpromazine
***
***
Supplementary Figure S2. Intracellular trafficking and internalization mechanisms of Nb-LNPs.
(A) Quantitative analysis of the relative cellular uptake of Nb-LNPs in DC2.4 cells. Cells were pre-treated with specific pharmacological endocytosis inhibitors, including chlorpromazine (an inhibitor of clathrin-mediated endocytosis), amiloride (an inhibitor of macropinocytosis), and filipin (an inhibitor of caveolae-mediated endocytosis), or incubated at 4°C (to block energy-dependent internalization). Data are presented as mean ± SD (n = 6). ***p < 0.001 compared to the untreated control group. Statistical significance was determined by one-way ANOVA.

## Slide 3
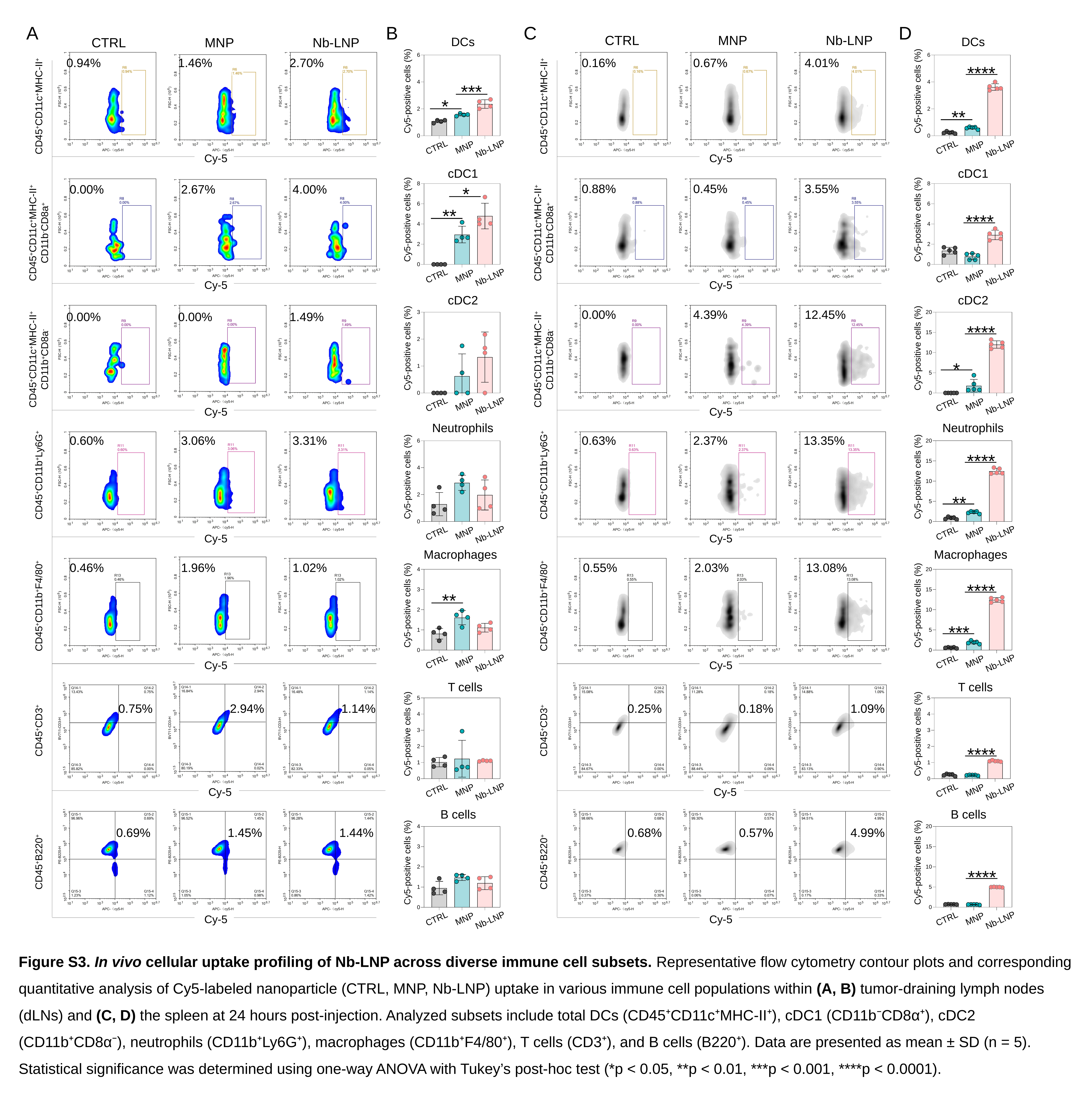

A
B
C
D
CTRL
MNP
Nb-LNP
DCs
CTRL
MNP
Nb-LNP
Cy5-positive cells (%)
CD45+CD11c+MHC-II+
CD45+CD11c+MHC-II+
CD11b-CD8a+
CD45+CD11c+MHC-II+
CD11b+CD8a-
CD45+CD11b+Ly6G+
CD45+CD11b+F4/80+
CD45+CD3+
CD45+B220+
Cy-5
Cy-5
Cy-5
Cy-5
Cy-5
Cy-5
Cy-5
0.16%
0.67%
4.01%
0.88%
0.45%
3.55%
0.00%
4.39%
12.45%
0.63%
2.37%
13.35%
0.55%
2.03%
13.08%
0.25%
0.18%
1.09%
0.68%
0.57%
4.99%
****
**
cDC1
CTRL
MNP
Nb-LNP
Cy5-positive cells (%)
****
cDC2
CTRL
MNP
Nb-LNP
Cy5-positive cells (%)
****
*
Neutrophils
CTRL
MNP
Nb-LNP
Cy5-positive cells (%)
****
**
Macrophages
CTRL
MNP
Nb-LNP
Cy5-positive cells (%)
****
***
T cells
CTRL
MNP
Nb-LNP
Cy5-positive cells (%)
****
B cells
CTRL
MNP
Nb-LNP
Cy5-positive cells (%)
****
CTRL
MNP
Nb-LNP
DCs
CTRL
MNP
Nb-LNP
Cy5-positive cells (%)
***
*
cDC1
CTRL
MNP
Nb-LNP
Cy5-positive cells (%)
*
**
cDC2
CTRL
MNP
Nb-LNP
Cy5-positive cells (%)
Neutrophils
CTRL
MNP
Nb-LNP
Cy5-positive cells (%)
Macrophages
CTRL
MNP
Nb-LNP
Cy5-positive cells (%)
**
T cells
CTRL
MNP
Nb-LNP
Cy5-positive cells (%)
B cells
CTRL
MNP
Nb-LNP
Cy5-positive cells (%)
0.94%
1.46%
2.70%
CD45+CD11c+MHC-II+
CD45+CD11c+MHC-II+
CD11b-CD8a+
CD45+CD11c+MHC-II+
CD11b+CD8a-
CD45+CD11b+Ly6G+
CD45+CD11b+F4/80+
CD45+CD3+
CD45+B220+
Cy-5
Cy-5
Cy-5
Cy-5
Cy-5
Cy-5
Cy-5
0.00%
2.67%
4.00%
0.00%
0.00%
1.49%
0.60%
3.06%
3.31%
0.46%
1.96%
1.02%
0.75%
2.94%
1.14%
0.69%
1.45%
1.44%
Figure S3. In vivo cellular uptake profiling of Nb-LNP across diverse immune cell subsets. Representative flow cytometry contour plots and corresponding quantitative analysis of Cy5-labeled nanoparticle (CTRL, MNP, Nb-LNP) uptake in various immune cell populations within (A, B) tumor-draining lymph nodes (dLNs) and (C, D) the spleen at 24 hours post-injection. Analyzed subsets include total DCs (CD45⁺CD11c⁺MHC-II⁺), cDC1 (CD11b⁻CD8α⁺), cDC2 (CD11b⁺CD8α⁻), neutrophils (CD11b⁺Ly6G⁺), macrophages (CD11b⁺F4/80⁺), T cells (CD3⁺), and B cells (B220⁺). Data are presented as mean ± SD (n = 5). Statistical significance was determined using one-way ANOVA with Tukey’s post-hoc test (*p < 0.05, **p < 0.01, ***p < 0.001, ****p < 0.0001).
